# Regional patterns of genetic structure and environmental differentiation in willow populations (*Salix humboldtiana* Willd.) from Central Mexico

**DOI:** 10.1002/ece3.5475

**Published:** 2019-08-02

**Authors:** Mariana S. Hernández‐Leal, Marco Suárez‐Atilano, Daniel Piñero, Antonio González‐Rodríguez

**Affiliations:** ^1^ Instituto de Investigaciones en Ecosistemas y Sustentabilidad Universidad Nacional Autónoma de México Morelia México; ^2^ Departamento de Ecología de la Biodiversidad, Instituto de Ecología Universidad Nacional Autónoma de México México City México; ^3^ Programa de Doctorado en Ciencias Biomédicas Universidad Nacional Autónoma de México México City México; ^4^ Departamento de Ecología Evolutiva, Instituto de Ecología Universidad Nacional Autónoma de México México City México

**Keywords:** ecological niche modeling, genetic diversity, genetic structure, niche divergence, riparian ecosystem

## Abstract

**Aim:**

To infer the geological and climatic factors that have shaped the genetic diversity and structure of a willow species (*Salix humboldtiana*) in three basins of Central Mexico.

**Location:**

Central Mexico.

**Methods:**

We collected samples from 11 populations across two hydrological basins (Balsas and Lerma) and one population from another basin (Ameca) within the Mexican Central Plateau (MCP). Individuals were analyzed using sequences of two chloroplast DNA (cpDNA) regions and eight nuclear simple sequence repeats (nSSR). Population genetic diversity and structure were determined from these data. To evaluate whether genetic structure was associated with ecological niche differentiation, we determined whether there is niche equivalence, overlap, or divergence between the Balsas and Lerma basins. Also, we evaluated the relative contributions of geographic distribution and climatic variation on population genetic structuring through redundancy analysis (RDA) and partial RDA.

**Results:**

Both cpDNA and nSSRs data indicated the presence of three highly differentiated genetic groups, mostly geographically congruent with the three main hydrological basins. According to nSSRs, the three genetic groups can be further subdivided into eight subgroups corresponding to different rivers within the main basins. The niche equivalency test showed that the niches of the species in the Balsas and Lerma basins are significantly nonequivalent. The RDA indicated a significant association of genetic variation among populations with climate variables (particularly those related to the precipitation regime), while controlling for geographic distribution.

**Main conclusions:**

The genetic structure of *S. humboldtiana* is strongly associated with the historical and current geological configuration of the basins and the rivers within basins. The observed hierarchical genetic differentiation can be due to gene flow limitation resulting from physical barriers to the dispersal of *S. humboldtiana*, but also to some degree of isolation by environment, as suggested by the significant association between genetic variation among populations and precipitation regime.

## 1 INTRODUCTION

The genetic structure of populations and the establishment of new evolutionary lineages are strongly mediated by processes associated with the exchange of genetic information within and among populations (Hamrick & Schnabel, 1985). Understanding how the environment affects the processes of diversification and genetic differentiation has been considered a fundamental aim in evolutionary biology (Kozak & Wiens, 2006; Pease et al., 2009; Wiens & Graham, 2005). In recent years, the use of ecological niche modeling (ENM) in combination with genetic data has allowed biologists to infer how the environment influences the differentiation among species and genealogical lineages (Alvarado‐Serrano & Knowles, 2014; Costion et al., 2015; Kalvik, Stout, Doonan, & Parkinson, 2012; Kozakn & Wiens, 2006). This kind of analysis has caused a debate on how the niches of closely related species evolve. For example, when two early divergent lineages that have passed to allopatric divergence (i.e., sister lineages) retain similar ecological traits, we refer to it as niche conservatism. On the other hand, environmental differences across a species' distribution may cause morphological and genetic differentiation on populations and also niche divergence among them, even without the presence of a physical barrier (Costion et al., 2015; Makowsky, Marshall, McVay, Chippindale, & Rissler, 2010; Peterson & Holt, 2003; Raxworthy, Ingram, Rabibisoa, & Pearson, 2007; Wiens & Harrison, 2004; Wiens & Graham, 2005). In widely distributed species, it may be difficult to distinguish if a physical barrier (niche conservatism) or adaptation to a new environment (niche divergence), or a combination of both, is responsible for the divergence. However, these two scenarios can be distinguished by an analysis of niche similarity combined with the assessment of the geographic distribution of genetic diversity. Previous analysis of divergence within widely distributed tree species with populations living in different environments has shown that climate can influence genetic and morphological variation (Riordan et al., 2016; Sork et al., 2010), what could lead to differentiation through local adaptation or simply because of variation in flowering phenology (Cavender‐Bares & Pahlich, 2009; Huang et al., 2015; Sork et al., 2016).

**Figure 1:**
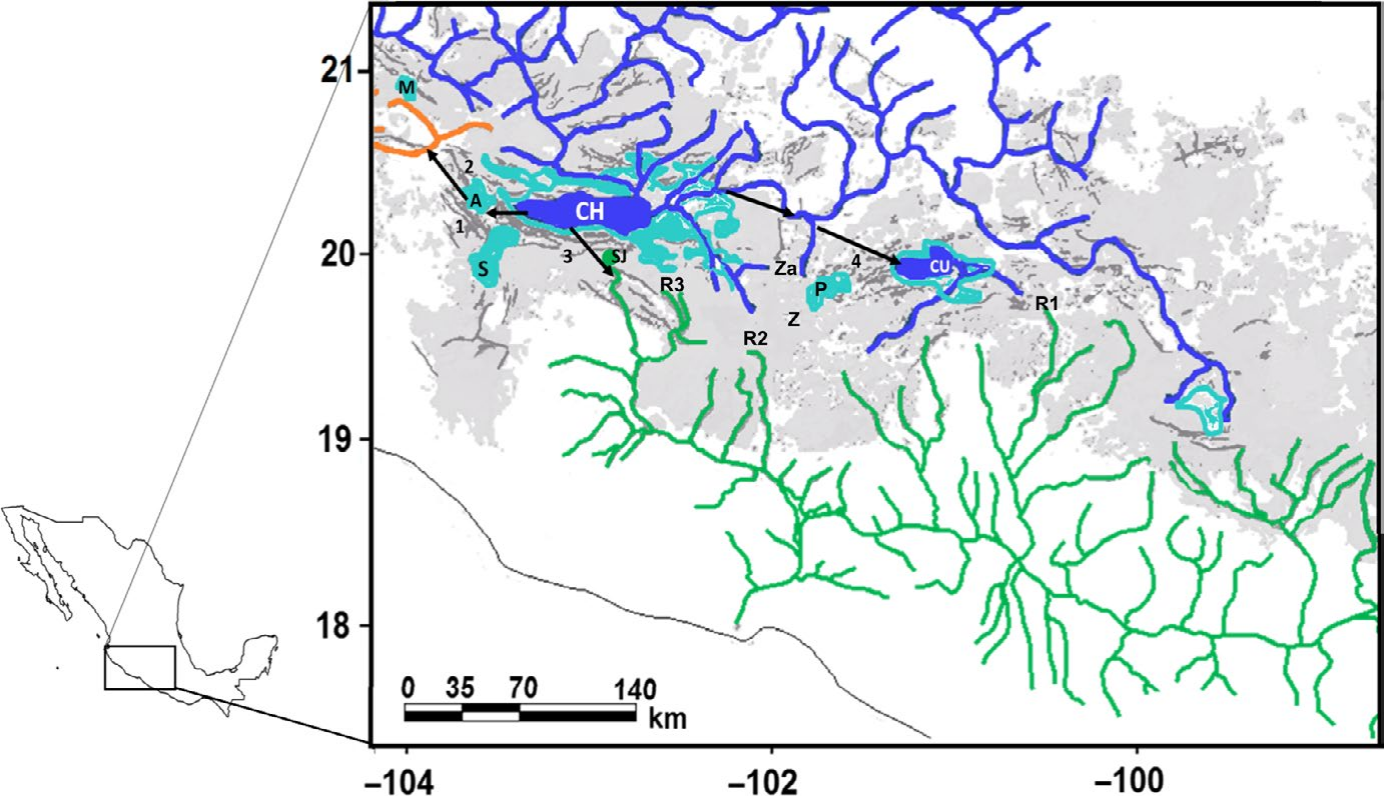
Main basins in the Mexican Central Plateau (MCP) and geological events. The Transmexican Volcanic Belt (TMBV) is represented in light gray. Miocene‐Pliocene paleolakes extension is represented in light blue and in bold blue their current size. Arrows show possible historical connections between basins. Lakes: S, Sayula; M, Magdalena; A, Zacoalco‐Ameca; SJ, Sn. Juanico; P, Pátzcuaro; CU, Cuitzeo; CH, Chapala; Z, Zirahuén; Za, Zacapu. Rivers: R1, Tuxpan; R2, Ario de Rosales; R3, Cupatitzio. Faults and trenches are represented by darker gray lines and numbers. 1, San Marco; 2, Ameca; 3, Chapala‐Tula; 4, Corredor Volcánico Tarasco. Color lines represent current water courses of the basins (green = Balsas; orange = Ameca; blue = Lerma) (modified from Domínguez‐Domínguez et al., 2008)

Central Mexico has a very complex geological and climatic history. One of the most prominent physiographical features within this area is the Mexican Central Plateau (MCP), a massive uplift that has been modified by the intense volcanism present since the Miocene, product of the activity of the Transmexican Volcanic Belt (TMVB; Tamayo & West, 1964). During the periods of volcanic activity, the lake basins and rivers that run along the MCP (mainly the river Lerma and tributaries of the Balsas and the Ameca) have suffered multiple events of fragmentation and connection (Ferrari, Orozco‐Esquivel, Manea, & Manea, 2012; Tamayo & West, 1964). Also, the climatic changes during the Pleistocene modified the extension and water levels of the lakes, during alternating humid and dry periods of the glacial cycles, producing events of connection and vicariance for the populations of associated species (see Figure 1). For example, there is paleolimnological evidence that a connection between the Ameca and Lerma rivers was possible through different lakes like Chapala and San Marcos. During some periods of the Pliocene and Pleistocene, these lakes were wider than today making the exchange of different species possible (Barbour, 1973; Tamayo & West, 1964). Other basins that were interconnected with the Lerma were the upper parts of rivers Tuxpan, Cupatizio, and Ario de Rosales, that now are part of the Balsas Basin (Álvarez, 1972; Tamayo & West, 1964). All these changes have greatly influenced the evolution of different aquatic taxa (Domínguez‐Domínguez, Alda, León, García‐Garitagoitia, & Doadrio, 2008; Domínguez‐Domínguez, Doadrio, & Pérez‐Ponce de León, 2006). Several phylogeographic studies have shown that many species of fish, principally of the family Goodeidae, and their parasites, have experienced multiple events of speciation product of these processes of connection and fragmentation between rivers (Corona‐Santiago, 2013; Domínguez‐Domínguez, Pérez‐Rodríguez, Escalera‐Vázquez, & Doadrio, 2009; Huidrobo, Morrone, Villalobos, & Álvarez, 2006; Mejia‐Madrid, Vázquez‐Domínguez, & Pérez‐Ponce De León, 2007; Pérez‐Rodríguez, Domínguez‐Domínguez, León, & Doadrio, 2009). Nevertheless, there is a lack of phylogeographic studies about how these changes have affected other species associated with rivers, like trees that form riparian forests.

Riparian forests are defined as the arboreal vegetation that grows near rivers, lakes, and floodplains. These ecosystems are characterized by a linear distribution of populations along the river, so they are also susceptible to changes in the distribution of watercourses (Mitsui, Isagi, & Setoguchi, 2010). However, contrary to fish fauna, whose dispersal patterns are confined to river courses, riparian plants usually can disperse their seeds and pollen through wind and water, enabling gene flow between populations of different rivers. Among riparian trees, willows (genus *Salix*) outstand as one of the most important ecological components of these ecosystems in boreal and temperate areas of the Northern hemisphere (Newsholme, 1992). Willows are usually dioecious trees or shrubs that discharge little plumed seeds primarily dispersed by wind and secondarily by water (Gage & Cooper, 2005; Karrenberg, Edwards, & Kollmann, 2002; Seiwa et al., 2008). These attributes make them an attractive model for studying the evolution and dynamics of freshwater ecosystems.

*Salix humboldtiana* is a diploid (2*n* = 38) riparian pioneer tree that can reach a height of up to 25 m. As the majority of willows, *S. humboldtiana* is an insect‐pollinated dioecious species that reproduces sexually by seed, but also shows frequent vegetative propagation through the establishment of branch fragments. It is the single *Salix* species distributed from the south of the United States to the Patagonia in Argentina in tropical and subtropical climates. In Mexico, it is commonly found from sea level up to 2,300 m, forming patches of riparian forest along river courses. *Salix humboldtiana* is commonly found in the Lerma and Balsas river systems within the MCP. Therefore, this species represents a good model to infer how topography and climate have affected the patterns of structure and gene flow of riparian forests in this region. The first goal of the present study was to determine the genetic structure of *S. humboldtiana* in two basins within the MCP using both nuclear and chloroplast DNA markers. Then, by combining genetic data, ecological niche modeling, and spatial analyses, we aimed at elucidating the consequences of historical changes in the distribution of the rivers. The specific questions addressed were as follows: (a) How is the genetic structure of *S. humboldtiana* related to the past or the current geological configuration of these two basins within the MCP? (b) Are climatic conditions in the two basins different and how is this related to the genetic structure within this species?

## 2 MATERIALS AND METHODS

Fully detailed methodology, sampling, molecular protocols, and species records are available in Appendix S1.

### 2.1 Sampling and laboratory procedures

Leaf tissue was collected from a total of 133 individuals representing 12 sampling sites located in the basins of the rivers Balsas and Lerma and one additional sampling site from the Ameca river basin (Table S1 in Appendix S1). At each site, a minimum distance of 15 m was kept between successive samples. Leaves were dried in silica gel and stored in paper bags. Genomic DNA was extracted from dried tissue using the cetyl‐trimethyl ammonium bromide protocol (CTAB) described by Doyle and Doyle (1987).

A total of 17 nuclear simple sequence repeats (nSSRs) markers previously designed for *Salix burjatica* (Barker, Pahlich, Trybush, Edwards, & Karp, 2003) and *Salix arbutifolia* (Hoshikawa, Kikuchi, Nagamitsu, & Tomaru, 2009) were assayed for amplification and polymorphism. Also, we tested 10 universal chloroplast simple sequence repeats (cpSSRs) designed by Weising and Gardner (1999). Parameters of the thermal cycling programs and primers selected to be used in *S. humboldtiana* are specified in Appendix S1 (PCR protocols). Amplification products were analyzed at the Roy J. Carver Biotechnology Center in the Illinois University using an ABI Prism 3730 xl capillary sequencer, with the Liz 500^®^ (Applied Biosystems) size standard. Resulting electropherograms were analyzed in peak scanner 1.0 (Perkin Elmer Applied Biosystems) for allele sizing. Only eight nSSRs and one cpSSRs were polymorphic for this species and were used for the whole sample (Table S2 in Appendix S1).

### 2.2 Phylogenetic analysis

To assess historical relationships among *S. humboldtiana* populations in the three basins, we sequenced 444 bp from the psbA‐trnH and the 5′‐trnS intergenic spacer regions of the cpDNA from four individuals from each basin (Ameca, Balsas, and Lerma) chosen to represent the main river systems within them (detailed amplification and sequence analysis are specified in Appendix S1). No further samples were included in this analysis since initial data revealed very limited polymorphism and only among‐basin variation.

We performed a phylogenetic analysis using the 12 psbA‐trnH (GenBank accession numbers MH919415–MH919426) and 5′‐trnS (GenBank accession numbers MH924999–MH925010) sequences of *S. humboldtiana* individuals. A *Salix babylonica* sequence (GenBank accession number EU750539.1) was used as outgroup. Alignment of the sequences was performed manually with BioEdit 7.0.5 (Hall, 1999), and then, the psbA‐trnH and 5′‐trnS matrices were concatenated with Mesquite 3.31 (Madison & Madison, 2002). Both markers displayed only length variation, and therefore, indels were considered as a fifth character state. Levels of genetic variation for each basin, that is, the number of segregating sites (S), the number of haplotypes (*h*), and haplotype diversity (*Hd*), were estimated in dnasp5.10.01 (Librado & Rozas, 2009). A maximum‐likelihood (ML) method was used to construct the phylogenetic tree in MEGA 7.0.18 (Kumar, Stecher, & Tamura, 2016) with 10,000 bootstrap replicates.

### 2.3 Genetic diversity and structure

Since *Salix* is well known to reproduce clonally by cuttings, a distance matrix was used to identify individuals with identical multilocus nSSRs genotypes using the program GenoDive v 2.0b23 (Meirmains & Van Tienderen, 2004). We detected 16 clonal individuals in a total of 133 samples. Individuals with identical genotypes were always found within the same river, and particularly in two sites, Queréndaro and Jiquilpan (see Appendix S1 and Table S1). To avoid biases in the genetic diversity and differentiation estimates, only a single individual for each clonal multilocus genotype was retained for further analysis resulting in a total of 117 unique genets.

The nSSRs data were analyzed with the program FreeNa v1 (Chapuis & Estoup, 2007) to estimate the frequency of null alleles. The expectation maximization algorithm (Demster, Laird, & Rubin, 1977) was used, and FreeNa was run with 1,000 bootstraps. This program was also used to estimate differentiation (*F*_ST_) values among populations with the method of Weir (1996) implementing the ENA correction to account for null alleles (Chapuis & Estoup, 2007). Hardy–Weinberg (HW) equilibrium for each loci and linkage disequilibrium for all pairs of loci were examined in GENEPOP v 4.07 using an exact test based on MCMC with 1,000 iterations and 200 batches. The false discovery rate (FDR) was used to eliminate significance by chance in multiple comparisons (Benjamini & Hochber, 1995). This method was preferred over Bonferroni corrections because it has been proven that it is more effective in rejecting type‐one errors (Verhoeven, Simonsen, & McIntyre, 2005). Expected (*H*_E_) and observed (*H*_O_) heterozygosity and the inbreeding coefficient (*F*_IS_) in each locality were estimated using the software ARLEQUIN v 3.5.1.2 (Excoffier & Lischer, 2010) with 10,000 permutations.

The STRUCTURE v. 2.3 (Pritchard, Stephens, & Donnelly, 2000) program was run with the nSSRs data set, to assign populations and individuals into differentiated genetic groups. To run the program, the following setup was used: *K* varied from 1 to 12 with 50 iterations for each *K*, with a burn‐in of 10^5^ followed by 10^6^ Markov chain Monte Carlo (MCMC) iterations. To determine the most probable *K* value from the analysis, the highest value of Δ*K* was calculated implementing the Evanno method (Evanno, Regnaut, & Goudet, 2005) in the online version of Structure Harvester v 0.6.9 (Earl & vonHoldt, 2012). Additionally, the spatial Bayesian clustering algorithm implemented in GENELAND v 3.1.4. (Guillot, Leblois, Coulon, & Frantz, 2009; Guilliot, Mortier, & Estoup, 2005) was used to delimit the genetic groups while simultaneously considering the geographical location of the samples. First, we performed 15 independent runs with a number of *K*_min_ = 1 to *K*_max_ = 12 to estimate the number of genetic groups. Then, we fixed *K* value at 8, the maximum number of possible genetic groups with a probability of each individual assigned to a genetic group higher than .6. We ran 50 additional independent runs and chose the 10 runs with the highest mean logarithms of posterior probability. Runs were performed with 1 × 10^6^ MCMC iterations and a thinning value of 1,000. The STRUCTURE and GENELAND runs were summarized using CLUMPP (Jakobsson & Rosenberg, 2007), and the results were plotted in DISTRUCT (Rosenberg, 2004).

Analyses of molecular variance (AMOVA) using both the infinite alleles and the stepwise mutation models (IAM and SMM, respectively) were performed with ARLEQUIN v 3.5.1.2 (Excoffier & Lischer, 2010) with 10,000 permutations. These analyses were based on different population groupings to understand the partitioning of genetic diversity at different levels (among and within populations, between the Balsas and Lerma basins, and according to the group assignment obtained with the programs STRUCTURE and GENELAND). Pairwise values of *F*_ST_ and *R*_ST_ among populations were also obtained as well as their significance levels.

### 2.4 Ecological niche modeling

To assess differentiation in the ecological niche between *S. humboldtiana* populations in the Balsas and Lerma basins, an ecological niche modeling (ENM) approach was used. We collected environmental data from 65 *S. humboldtiana* spatially unique presence records from our own sampling and from botanical collection records (www.conabio.gob.mx/remib/doctos/remib_esp.html). To avoid biases resulting from unequal sampling across the area, we only considered records that were separated at least 10 km from each other. Final analyses included 24 records from the Balsas Basin and 33 from the Lerma Basin. To reduce the overprediction of area suitability as well as to perform a better model validation (Barve et al., 2011), we first defined an area of accessibility (sensu Soberón, 2007), considering the consensus hydrographic basins of Mexico (Priego‐Santander, Moreno Casasola, Palacio Prieto, López Portillo, & Geissert Kientz, 2003) to select those polygons assigned to the Balsas and Lerma basins.

For the ENM analysis, the 19 bioclimatic variables from the World Clim database were used (http://www.worldclim.org/bioclim; Hijmans, Cameron, Parra, Jones, & Jarvis, 2005), as well as one topographic variable (slope) derived from the elevation grid from the Hydro1k database (https://webgis.wr.usgs.gov/; U.S. Geological Survey, 2001), with a resolution of 30” (≈1 km^2^). To avoid over‐parameterization of the models, we performed Pearson's correlation tests among all pairs of variables. From each pair of highly correlated variables (*r* ≥ .8), we chose the more temporally inclusive one (e.g., preferring annual mean temperature over mean temperature of the warmest quarter). These analyses were performed with XLSTAT Pro 7.5 (www.xlstat.com/en/products-solutions.html). As a result, 10 climatic variables (Table 3B) and slope were selected. Slope has been regarded as an important factor for seed and juvenile establishment in many willow species (Azami et al., 2013).

The maximum entropy algorithm implemented in MAXENT 3.3.3 (Phillips, Anderson, & Schapire, 2006) was used for ENM. Analyses were performed with a convergence threshold of 1 × 10^–5^ with 500 iterations. Because MAXENT produces maps in a logistic probability format (from 0 to 1), we converted each model into binary (presence–absence or 1–0) format using as a threshold the 10‐percentile training presence, that is, the probability assigned to the 10th percentile of training occurrence records (Pearson, 2007). We used 75% of occurrence records from each basin for model training and 25% for model testing. To evaluate overall classification accuracy in the model performance, MAXENT estimates the area under the curve (AUC) from the receiver operating characteristic (ROC) plot (Phillips et al., 2006). We used an alternative and external transformation of this AUC into partial‐ROC graphics, as suggested based on AUC drawbacks (Lobo, Jiménez‐Valverde, & Real, 2008; Peterson, Papeş, & Soberón, 2008). The statistical significance of partial‐ROC graphics, and thus the AUC, was assessed using null distributions of expectations created via 1,000 bootstrap replicates of 50% of the total available points and calculating the probability that the mean AUC ratio was ≤1.

To evaluate whether the ecological niches of populations in the Lerma and Balsas basins are equivalent, we reciprocally projected each of the two models into the other's geographical space; that is, the model of the Balsas Basin was projected into the Lerma area and vice versa (Appendix S2 and Figure S1). Then, we estimated the overlap index between the two niche models (OI). This index represents the fraction of the niche model of one basin predicted by the other (Martínez‐Gordillo, Rojas‐Soto, & Espinosa de los Monteros, 2010). Second, to determine whether the ecological niches from both basins were significantly different and whether their niche spaces were interchangeable, we used a modification of the method proposed by Warren, Glor, Turelli, and Funk (2008). The niche similarity and equivalency tests are directly performed from environmental space with no need to build an ENM. These statistics allow the identification of environmental differences between population groups (Broenimann et al., 2011; Petipierre et al., 2012). The first test estimates the empirical values of overlap between ecological niches (environmental spaces), generating values of niche overlap (*D*) and niche similarity (*I*) with ranges from 0 (no overlap) to 1 (identical niche models). The second test, the niche equivalency (or identity), determines whether the niches of, in this case, the populations in the two basins are more different than expected by chance even if they are drawn from the same underlying distribution. The latter was done based on *D* and *I* values randomly generated by 100 pseudo replicates and compared against empirical values with a one‐tailed *t* test (Warren, Glor, & Turelli, 2010; Warren et al., 2008). Finally, we evaluated the background similarity, an analysis that incorporates environmental heterogeneity to test whether the observed niche models with partially or entirely nonoverlapping distributions are any more different from one another than expected by chance (null hypothesis), or as a result of differences in habitat suitability or selection (Glor & Warren, 2010; Warren et al., 2008). The modified method estimates the use of the environment with a density of occurrence layer in environmental space, which is the result of a smoothed kernel density function of observed occurrences (Broenimann et al., 2011).

We used the Ecospat package in R to estimate the density of occurrences and to compare the environmental spaces of populations in the Balsas and Lerma basins. A density Gaussian kernel was constructed from the given set of occurrences in environmental space in both basins following Broenniman et al. (2011). The Kernel function was used with a band width equal to 0.9 times the minimum of the standard deviation and the interquartile range of the data divided by 1.34 times the sample size to the negative one‐fifth power (Silverman, 1986). We set the resolution of environmental space to a graticule of 100 × 100 pixels, so that each environmental pixel would be defined by approximately a 60‐mm interval in precipitation and a 0.3°C interval in temperature (assuming these variables were completely related to orthogonal components). One hundred and 1,000 iterations were performed to test niche equivalency and niche similarity, respectively. Significance was evaluated by the position of the observed value in relation to the distribution of simulated values under the null models of each hypothesis.

Finally, to determine whether geographic distance, climatic variation, or both have an influence on the genetic structuring among populations, we carried out redundancy and partial redundancy analyses (RDA and pRDA, respectively) using the “vegan” library in R (Oksanen, 2015). The environmental variables chosen for this analysis were the 10 climatic variables and slope used in the ENM analyses (Table 3B). As genetic variables, we used the individual scores on the first two axis of a principal coordinates analysis (PCoA) performed in GenAlex v 6.5 (Peakall & Smouse, 2006, 2012) from the genotypes at the eight nSSR loci. The spatial coordinates from each individual were used as geographic variables. We ran three different models, a full model including both geographic and environmental explanatory variables, and two partial models, firstly using only environmental variables and controlling for geographic effects (pRDA1), and secondly including geographic variables and controlling for environmental effects (pRDA2).

## 3 RESULTS

### 3.1 Phylogenetic analysis

**Figure 2:**
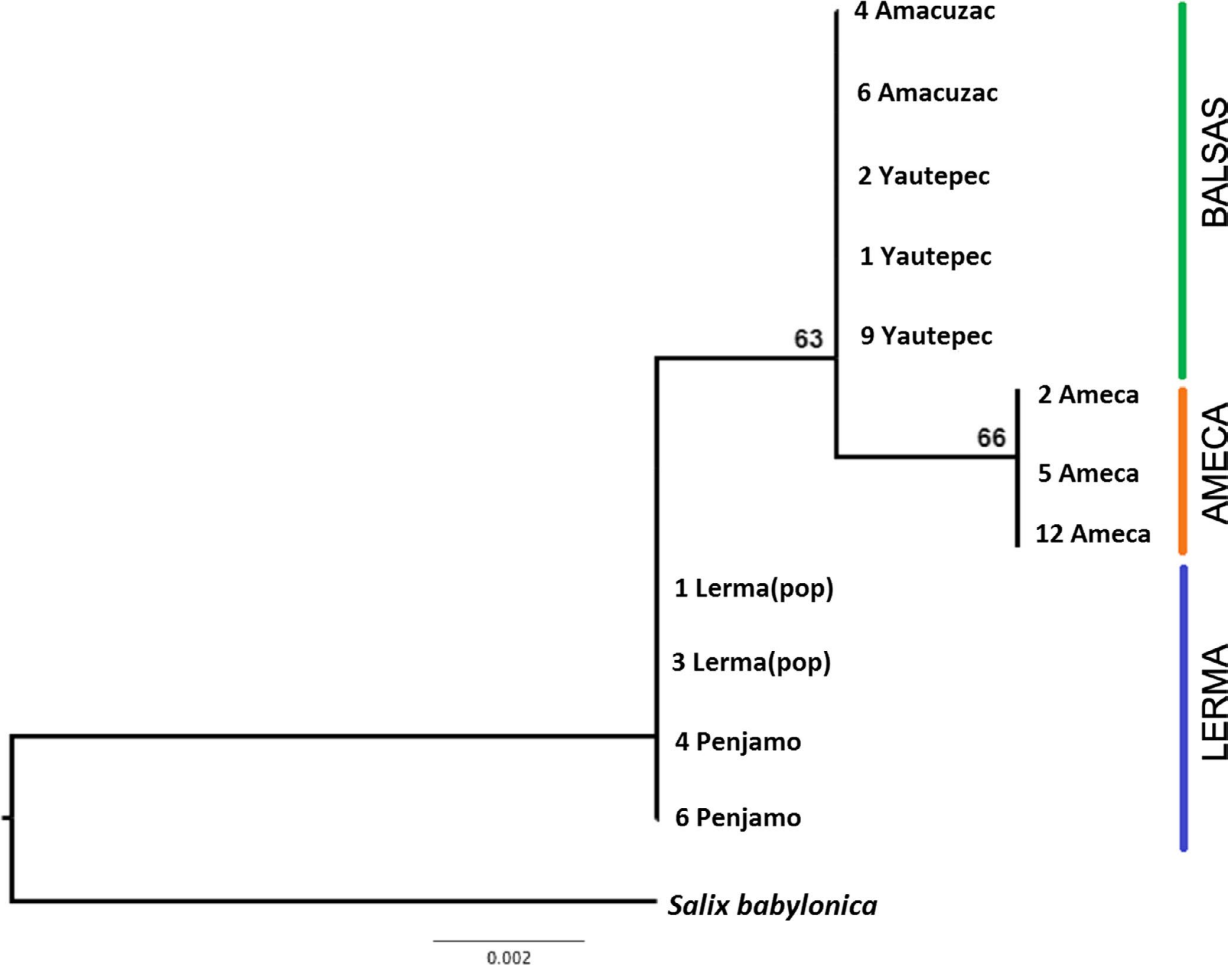
Phylogenetic reconstruction of *S. humboldtiana* haplotypes using cpDNA sequences of psbA‐trnH and 5′‐trnS. Colored lines refer to the three different basins (green = Balsas; orange = Ameca; blue = Lerma). Codes are composed by individual number and population of origin. Bootstrap values are shown only for Ameca and Balsas clades. Consensus tree based on 1,000 trees

A total of 444 bp were aligned from chloroplast sequence data. Sequence variation corresponded only two indels that defined three different haplotypes. Total haplotype diversity was moderate (*Hd* = 0.691). Each of the three haplotypes corresponded to one of the basins, Ameca, Balsas, and Lerma (Figure 2).

### 3.2 Genetic diversity and structure

**Figure 3:**
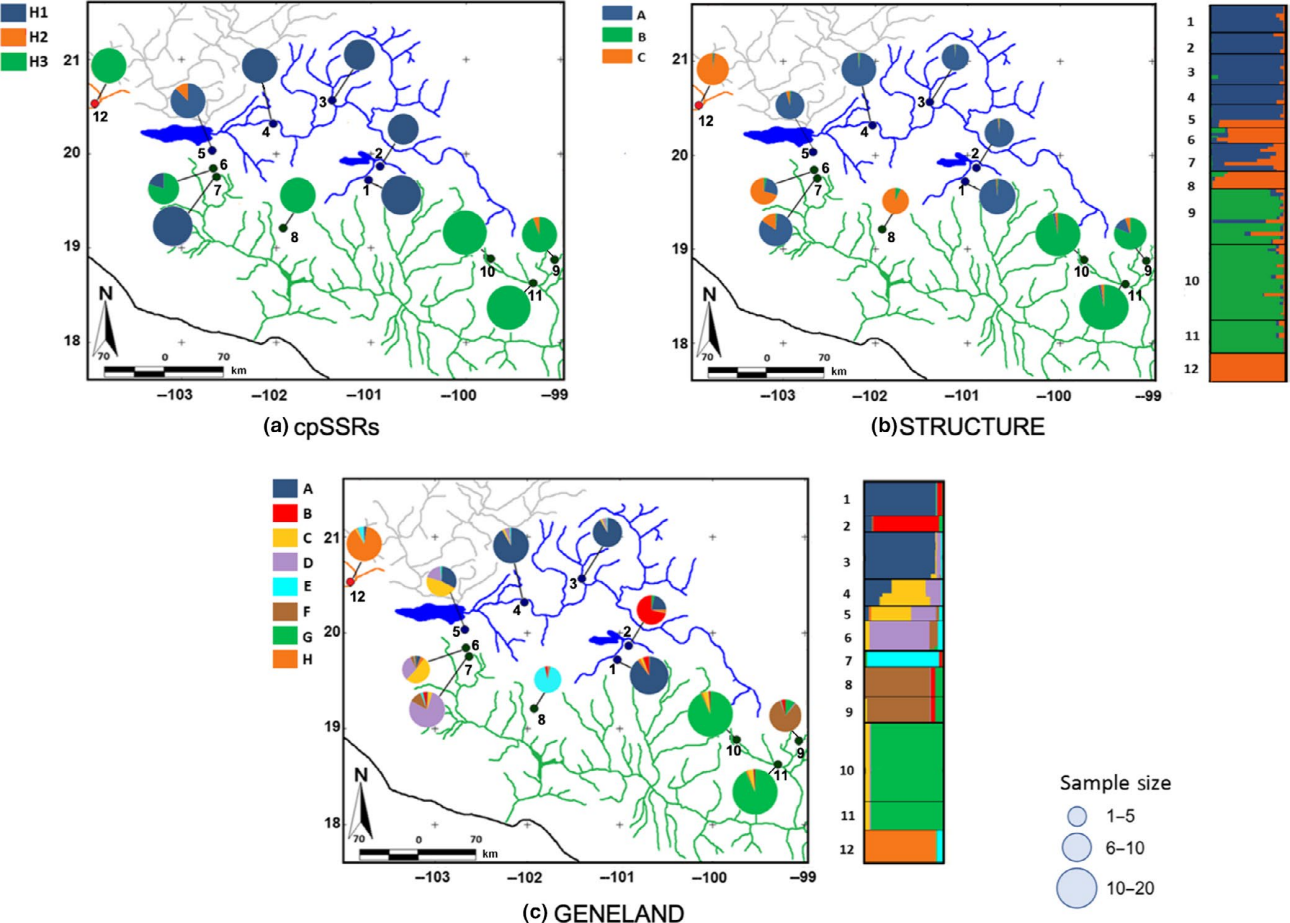
Geographic structure of genetic variation in one cpSSR and eight nSSR loci in 12 populations of *S. humboldtiana*. Colored lines indicate sampled basins in this study (green = Balsas; orange = Ameca; blue = Lerma). Gray lines represent the Santiago Basin (part of the Lerma‐Santiago system), but not included in this study. In each panel, colored pay charts represent the genetic composition of the populations, with size of the circle proportional to sample size after removing clones. (a) Frequency of three alleles (H1, H2, H3) at a cpSSRs locus. (b) STRUCTURE and (c) GENELAND proportion of assignment to each genetic group within each sampling locality based on eight nSSRS. Assignment is represented at both the population (pay chart) and individual (right bar plot) levels

Eight nSSRs and one cpSSR were polymorphic for *S. humboldtiana *(Table S2 in Appendix S2). In the cpSSR, we found only three different alleles whose frequencies in each population are shown in Figure 3a. The H1 allele was found in all populations sampled in the Lerma Basin and in two populations (6 and 7) from the headwaters of the Balsas Basin. H2 was rare and only found in a few individuals from the Lerma and the Balsas basins, but from widely separated populations. Finally, H3 was found in most populations from the Balsas Basin and in the single population sampled in the Ameca Basin.

For the eight nSSRs, we found 101 alleles, 28 of which are private alleles to one locality (Table 1). According to the FreeNA analysis, null alleles are present in two populations (Yautepec and La Parota; Table S3 in Appendix S2), but the ENA correction did not indicate an overall effect product of null alleles on the calculation of *F*_ST_ (not using ENA = 0.269, using ENA = 0.263; Table S4 in Appendix S2). There were statistically significant deviations from Hardy–Weinberg equilibrium due to heterozygote deficiency in the populations Ameca (loci Cha 437 and Sb 233), Yautepec (Cha 475, Cha 528, Sb 201, Sb233), B. Amacuzac (Cha 475, 433, 437 and Sb 233), and La Parota (all loci except, Sb 201). Mean values of the expected (*H*_E_) and observed (*H*_O_) heterozygosity, inbreeding coefficient (*F*), number of alleles per locus (Na), number of effective alleles per locus (Ne), allelic richness (Ar), and number of private alleles (Pa) per population are listed in Table 1.

**Table 1:** Genetic diversity in 12 populations of *S. humboldtiana*

ID	Pop	Basin	*N*	Na	Ne	Ar	Pa	*H*_E_	*H*o	*F*
1	Charo	L	9	5	3.5	4.09	3	0.78	0.79	0.048
2	Queréndaro	L	6	3.9	3.2	3.69	1	0.742	0.857	−0.243
3	Lerma	L	9	5	3.8	4.34	1	0.768	0.653	−0.057
4	Penjamo	L	6	4.9	3.5	4.47	4	0.703	0.667	−0.082
5	Jiquilpan	L	8	4.9	3.6	4.28	2	0.723	0.719	−0.411
6	Sn. Juanico	B	5	3.9	2.9	3.87	1	0.631	0.65	−0.224
7	Tocumbo	B	9	5.1	3.6	4.30	2	0.734	0.667	−0.001
8	La Parota	B	5	1.6	1.5	1.62	1	0.361	0.1	0.905 HD
9	Yautepec	B	17	4.6	2.4	3.08	5	0.534	0.354	0.712 HD
10	B. Amacuzac	B	19	3.5	2.0	2.77	4	0.502	0.323	0.092 HD
11	A. Amacuzac	B	15	3.7	2.4	2.66	2	0.521	0.482	−0.188
12	Ameca	A	9	3.5	2.3	2.99	2	0.543	0.407	0.629 HD
	Total		117	101	4.146	5.061	28	0.778	0.5	0.36

Note

HD: Hardy–Weinberg disequilibrium (significant heterozygote deficit; *p* < .05).

Abbreviations: Ar: allelic richness (rarefacted); Basin: L: Lerma, B: Balsas, A: Ameca; *F*: inbreeding coefficient; *H*_E_: expected heterozygosity; *H*_o_: observed heterozygosity; ID: identification number of population; *N*: number of individuals analyzed; Na: mean number of alleles per locus; Ne: mean number of effective alleles per locus; Pa: number of private alleles; Pop: population name.

Populations with significant heterozygote deficit were evaluated with the INest v2.2 software (Chybicky & Burczyk, 2009) to separate actual inbreeding from the effect of null alleles on deviations from Hardy–Weinberg equilibrium. For populations, La Parota, B. Amacuzac, and Sn Matute inbreeding is the significant component of the model, while in the case of Yautepec, null alleles seem to have more weight (Table S5 in Appendix S2).

We identified *K* = 3 in STRUCTURE as the most probable number of clusters after calculating Δ*K* (Figure 3b). These clusters greatly corresponded to the Lerma Basin and upper part of west Balsas (blue cluster), upper part of east Balsas and southeastern Balsas (green cluster) and Ameca, and upper part of west Balsas (orange cluster). In the spatially explicit analyses performed in GENELAND, the most probable number of clusters was *K* = 8 (Figure 3c). These eight clusters presented a more detailed spatial grouping of populations. In both STRUCTURE and GENELAND analyses, individuals corresponding to the populations 6 (Tocumbo) and 7 (Sn. Juanico) that belong to the upper west part of the Balsas Basin revealed a similar genetic composition to individuals from location 5 (Jiquilpan) situated in the southwestern portion of the Lerma Basin (Figure 3).

The results of the AMOVAs for the different groupings are shown in Table 2. The analyses with the IAM (*F*_ST_) indicated that 13.65% of the variation is found among the Balsas and Lerma basins, while 18.41% is among populations within basins, and 67.94% is within populations. In turn, the grouping of individuals according to the three genetic groups identified by STRUCTURE indicated that 18.7% of the variation is among groups, 12.93% among populations, and 68.38% within populations. Finally, the grouping according to the eight genetic groups identified by GENELAND indicated that 21.38% of the variation is among the genetic groups, 7.37% among populations, and 71.25% within populations. The analysis with the SMM (*R*_ST_) showed similar trends, but the proportion of the genetic variation explained at the among‐groups level was considerably higher, indicating that mutations are contributing to the differentiation among population groups. Pairwise genetic differentiation values using *R*_ST_ and *F*_ST_ were statistically significant (*p* < .05) for almost all pairs of populations and ranged between 0.036 and 0.524 for *F*_ST_ and between −0.01 and 0.846 for *R*_ST_ (Table S6 in Appendix S2).

**Table 2:** Results of analyses of molecular variance (AMOVA) for *Salix humboldtiana* in central Mexico based on eight nSSRs loci with different population groupings: among and within all populations and grouped according to basin (Balsas/Lerma) and according to the results of the Bayesian clustering algorithms GENELAND and STRUCTURE

Grouping	*df*	*R*_ST_			*F*_ST_		
		SS	VC	%	SS	VC	%
Basin							
Among groups	1	44,098.3	354.7	28.9***	66.8	0.5	13.6***
Among populations within groups	10	75,334.8	368.5	30.1***	139.3	0.6	18.4***
Within populations	222	111,525.5	502.4	41	505.1	2.3	67.9
Total	233	230,958.6	1,225.5		711.2	3.3	
Structure							
Among groups	2	85,404.2	531.2	44***	113.6	0.6	18.7***
Among populations within groups	9	34,029	173.8	14***	95.2	0.4	12.9***
Within populations	222	111,525.5	502.4	42	509.2	2.3	68.4
Total	233	230,958.6	1,207.4		718.0	3.4	
Geneland							
Among groups	7	115,947.5	564.5	50.9***	178.8	0.7	21.38***
Among populations within groups	4	4,552.5	32.4	2.9***	27.3	0.2	7.37***
Within populations	222	112,631.4	513	46.2	505.2	2.3	71.25
Total	233	233,232.3	1,109.9		711.2	3.2	
Populations							
Among populations	11	120,499.9	543.7	51.8***	208.8	0.87	27.47***
Among individuals within populations	222	112,631.4	512.9	48.3	509.194	2.32	72.52
Total	233	233,131.3	1,063		711.2	3.1	

Note

Analyses were performed with both the infinite alleles (*F*_ST_) and stepwise (*R*_ST_) mutation models.

Abbreviations: *df*, degrees of freedom; SS, sum of squares; VC, variance components.

*** *p* < .001.

### 3.3 Ecological niche modeling and niche differentiation

**Figure 4:**
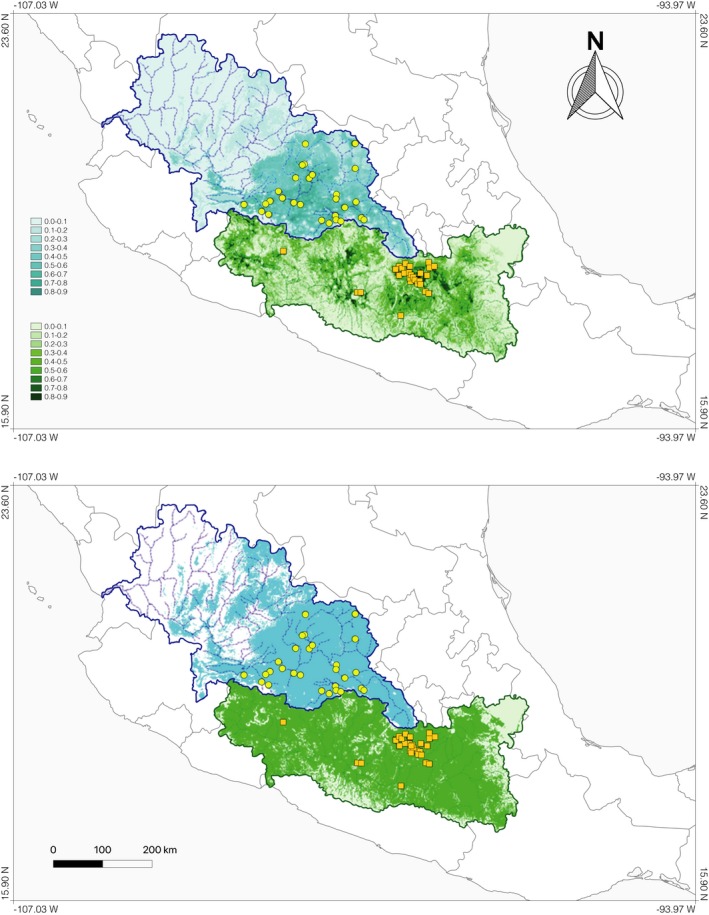
Ecological niche models (ENMs) obtained with MAXENT for *Salix humboldtiana* in basins Lerma = blue and Balsas = green. The map on the upper panel shows the logistic probability of environmental suitability. Darker areas represent higher probability of species occurrence. Circles and squares represent unique records of *S. humboldtiana* for the two basins obtained from REMIB data. ENMs were obtained using record data that were at least 10 km separated from each other (Balsas = 24) (Lerma = 32). Map on the lower panel shows the transformation of suitability probability into a binary (presence–absence) map using as a decision threshold the 10% of training presence

The performance of the two models (corresponding to the Balsas and Lerma basins) produced by MAXENT was significantly better than expected by chance alone according to the partial‐ROC bootstrap tests (Figure 4a), which showed significant ratio values of empirical AUC over null expectations (Balsas = 1.742, *p* < .001; Lerma = 1.714, *p* < .001). Regarding estimates of relative contributions of the environmental variables to the MAXENT models, results indicated that the topographic variable (slope) and the precipitation of the warmest quarter (Bio 18) were highly informative for both Balsas and Lerma models (slope = 45.3 and 14%, Bio 18 = 7.5 and 37%, respectively). Precipitation of the wettest quarter (Bio 16 = 28.9%) resulted highly informative for the Balsas model, while temperature seasonality was informative for the Lerma model (Bio 4 = 25%).

Our results indicated that the niche models for the two basins exhibited an overlap index of 54.37 for the Balsas to Lerma comparison and 45.62% for the Lerma to Balsas comparison (Appendix S2). Interpredictability (IP) was 90.62% considering the Balsas to Lerma comparison, while the totality of the Balsas records was predicted by the Lerma model (IP Lerma to Balsas = 100%).

**Figure 5:**
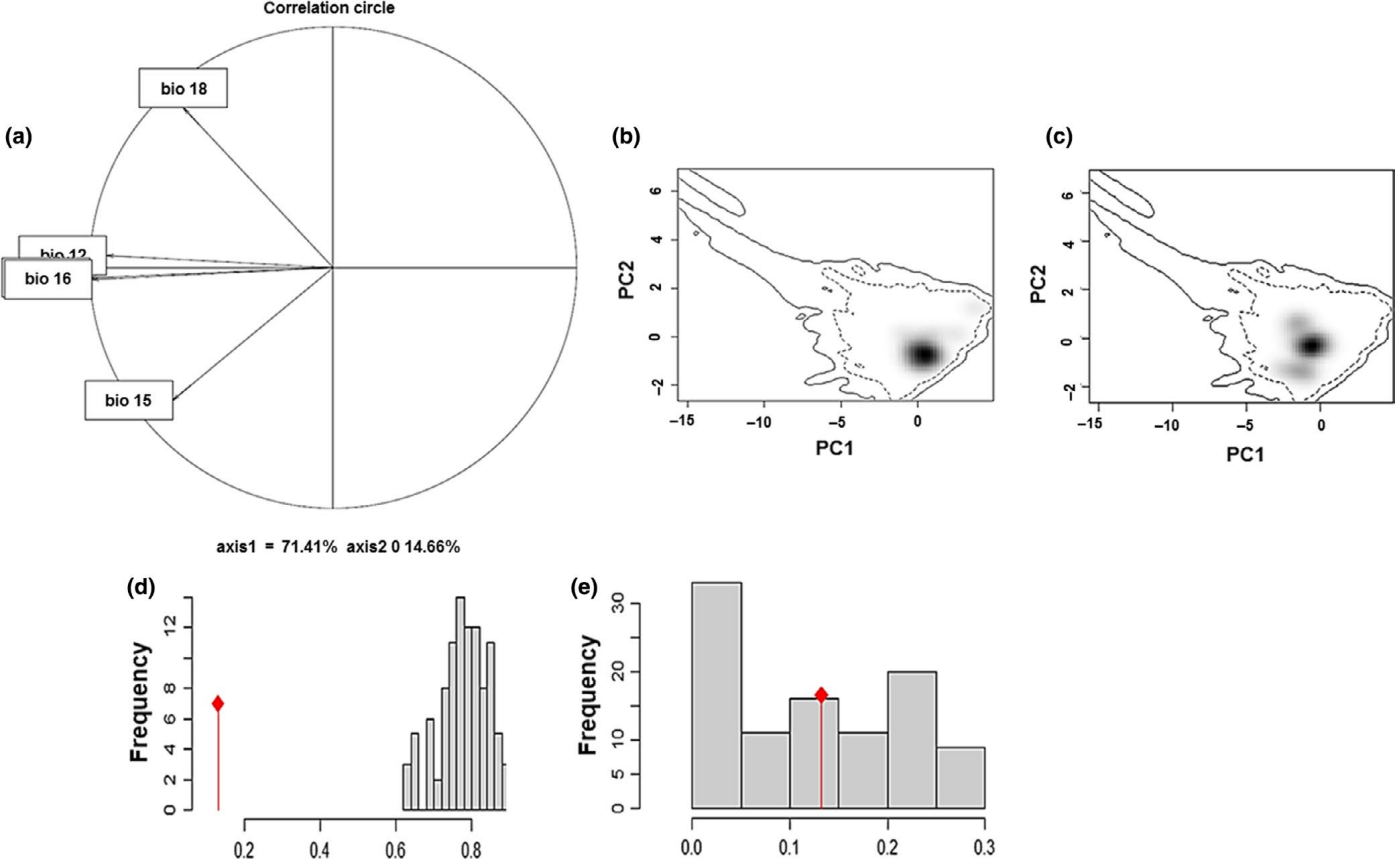
Differentiation of niche space between the Lerma and Balsas basins. (a) Correlation circle and the direction of the arrows indicate the correlation of each variable to each of the two principal components. (b) Occurrence density surfaces for the Lerma Basin and c) the Balsas Basin. The shaded area represents the density of environmental space. Niche equivalency (d) and niche similarity (e) test results. The similarity between two probability surfaces, measured by Hellinger's *D*, is shown. Values of *D* on the *x*‐axis and the corresponding histogram represent the expected distribution based on the randomization of the data under different null models. The first null model randomizes the identities of the records from the two basins (equivalency test), and the second one (similarity test) randomizes the distribution of the samples of each of the two basins within their accessibility area. The observed *D* value is shown by the red diamond

Results of the principal components analysis showed that ~85% of the environmental variation is explained by the first two principal components. All 10 climatic variables (previously chosen) and the slope were negatively correlated to the first PCA axis. Precipitation seasonality (Bio 15) was correlated positively to the second PCA axis, and precipitation of the warmest quarter (Bio 18) was negatively correlated to the second axis (Figure 5a). The occurrence density surfaces in environmental space indicated a separation in the environmental spaces of the populations in the two basins in at least one direction along the first PCA axis (Figure 5b,c, shaded areas).

The niche equivalency test showed that the niches of the Balsas and Lerma basins are not identical, showing a low similarity value of *D* = 0.134 (*p* = .02; Figure 5d). Furthermore, the niche similarity test showed the environmental spaces of both basins were not more similar than expected under the null model (Figure 5b,c; *D* = 0.4, *p* = .455).

### 3.4 Redundancy analysis test climatic and genetic association

The full RDA model considering the effect of both geography and climate on genetic structuring among populations was significant (*p* = .001) and accounted for a considerable proportion of the variance (*R* ^2^ = 57.3%; Table 3A). The pRDA1 for the association between genetic variation and climate while controlling for geography was also significant (*R* ^2^ = 21%, *p* = .001). In this analysis, the most important variables were annual precipitation and precipitation of the wettest month, followed by precipitation of the warmest quarter, temperature mean diurnal range, and slope (Table 3B). The pRDA2 indicated that geography alone explained a much smaller proportion of genetic variation but was still significant (*R* ^2^ = 4%, *p* = .003). Finally, climate and geography together accounted for 25% of the variation (*p* = .001).

**Table 3:** (A) Results of redundancy analyses (RDA) for association between genetic variation, geography, and climate for *Salix humboldtiana* populations. Two partial analysis are shown pRDA1 (effect of climatic variables while controlling for geographic effects) and pRDA2 (effect of geographic distribution while controlling for climatic variation). The proportion of variance explained by both geographic and climatic effects is also indicated (climatic/geographic). Proportion constrained corresponds to the partitioned variance relative to the constrained variance of the full RDA model. (B) Loadings of climate variables into the first two axes of pRDA1 and their significance

Microsatellite genetic variation	Partitioned variance	Proportion	*p*
(A)			
Full model: geography and climate (constrained variance)	0.08024	1	.001
pRDA1	0.01646	0.2052	.001
pRDA2	0.003281	0.04089	.002
Climatic/geographic	0.060499	0.24609	NA

Variable	*df*	pRDA1		
		Axis 1	Axis 2	*F*
(B)				
Bio 2	1	0.21	0.33	5.4***
Bio 3	1	−0.14	−0.31	2.3
Bio 4	1	0.20	0.22	3.2
Bio 7	1	0.22	0.36	1.02
Bio 12	1	0.12	−0.23	14.8***
Bio 13	1	0.42	−0.15	13.5***
Bio 15	1	0.42	0.12	2.4
Bio 16	1	0.31	−0.17	0.72
Bio 18	1	−0.07	−0.21	7***
Bio 19	1	−0.05	−0.17	0.31
Slope	1	−0.02	−0.25	5.1***

*** *p* = .0001; **.001; *.01.

## 4 DISCUSSION

In the present study, we found a hierarchical pattern of genetic structure among populations of *S. humboldtiana* at regional and local scales in the MCP. Regional scale differentiation could be explained by the historical and current geological and hydrological configuration of the main basins, Balsas, Lerma, and Ameca. Local structure is probably related to population dynamics inside watersheds and rivers where populations are found. Finally, evidence of ecological differentiation may suggest the influence of scenopoetic variables, mainly topography and precipitation, on the genetic structure and differentiation of *S. humboldtiana* populations located in these basins.

### 4.1 Geological history and genetic differentiation

Both nuclear and chloroplast DNA data are congruent with the idea that *S. humboldtiana* populations in the study area have experienced several vicariant events (Figures 2 and 3). There have been multiple connections and disconnections between rivers within and between basins at different times that have been important for delimiting gene flow among populations of organisms associated with water courses (Domínguez‐Domínguez et al., 2008; Mejia‐Madrid, Domínguez‐Domínguez, & Pérez‐Ponce De León, 2005; Rosas‐Elguera et al., 2003; Smith & Miller, 1986). For example, Domínguez‐Domínguez et al. (2008) found that the divergence between two main lineages of *Zoogoneticus quitzeoensis* (Goodeidae) distributed in an east–west gradient in the MCP occurred ca. 3.3 Mya based on the molecular clock calibration and was associated with the disconnection of water bodies during a dry period and the formation of geological isolation barriers. But dispersal of these organisms is restrained to river courses, and these connections have never been examined in an organism that is dispersed by both wind and water. Our results indicate very restricted gene flow among *S. humboldtiana* populations situated in different basins. Phylogenetic analysis of the psbA‐trnH and 5′trnS sequences revealed only three haplotypes, each restricted to one of the three basins, with a closer relationship between the haplotypes in the Ameca and Balsas basins (Figure 2). We must take this result with caution because of the reduced number of individuals used (12 individuals of *S. humboldtiana)* and the scarce genetic variation found. A similar pattern was obtained, though, from nuclear and chloroplast SSRs from all individuals, supporting in general a clear genetic differentiation among basins, with only a few populations showing evidence of admixture among basins. Particularly, in the case of the cpSSR locus, populations Tocumbo (6) and Sn. Juanico (7) belonging to the Balsas Basin, showed the haplotype present in all populations of the Lerma Basin, and population Ameca (12, Ameca basin) was fixed for the same haplotype present in almost all populations in the Balsas Basin (Figure 3a). For nuclear markers, Bayesian assignment into three genetic groups also indicated the sharing of genetic variation involving populations 5, 6, 7, 8, and 12 (Figure 3b,c).

These patterns are probably related to an ancient connection between the Chapala‐San Marcos and Zacoalco‐Atotonilco lake systems (Figure 1; Smith & Miller, 1986). It has been proposed that many headwaters that currently form the Balsas Basin were originally part of the Lerma. This is the case for the Tuxpan, Cupatitzio, and Ario de Rosales rivers, which were captured by the Balsas in different geological events (Rosas‐Elguera et al., 2003; Tamayo & West, 1964). The connections between hydrological systems are common, particularly in headwaters, where erosion and volcanic activity have changed the water course from one basin to another (Burr & Page, 1986; Kozak, Wiens, & Pfennig, 2006). These phenomena have been widely documented in the MCP rivers (Corona‐Santiago, 2013; Doadrio & Domínguez, 2004; Domínguez‐Domínguez et al., 2006; Domínguez‐Domínguez, 2009; Mateos, Sanjur, & Vrijenhoek, 2002; Smith & Miller, 1986; Webb et al., 2004). It has been stated that Sn. Juanico Lake was part of the ancient Chapala Lake, but during the Plio‐Pleistocene, an intense volcanic activity isolated part of the Chapala Lake that today is recognized as a part of the Balsas Basin (Álvarez, 1963; Barbour, 1973).

### 4.2 Genetic diversity and within basin differentiation

Levels of genetic variability in *S. humboldtiana* are similar to those found in other *Salix* species using the same nSSRs markers (Baker et al., 2003; Kikuchi, Suzuki, & Sashimura, 2011). Nevertheless, four populations showed significant levels of heterozygote deficit. High values of inbreeding could be associated with a small effective population size resulting from a high proportion of clonal individuals or an unequal sex ratio (Ueno, Suyama, & Seiwa, 2007; Crawford & Balfour, 1990). But also, a high frequency of null alleles in certain loci could inflate inbreeding values. *Salix humboldtiana* is dioecious and therefore an obligate outcrossing species, and even though it can reproduce by asexual propagation, our results indicated a relatively low frequency of clones. After analyzing genotypes with the Bayesian algorithm implemented in INEsT, significant inbreeding was detected in three populations (Ameca, B. Amacuzac, and La Parota) and only Yautepec showed a high frequency of null alleles (Table S3 in Appendix S2). Therefore, it seems likely that inbreeding in this study system is resulting from low effective population sizes associated with the frequent extinction and founding of populations. Like other riparian tree groups, willows are pioneer species with short generation times (no more than 20 years; Karrenberg et al., 2002; Liotta, 2001). Short life span in riparian ecosystems is related to a metapopulation dynamics in which populations establish in streambeds that are eroded periodically by water, causing recurrent extinction and establishment of populations along rivers (Karrenberg et al., 2002; Liotta, 2001; Oliveira‐Filho, Vilela, Gavilanes, & Carvalho, 1994). Usually, new populations are founded by few individuals, which could lead to frequent local inbreeding and genetic drift. These patterns have also been found in other willows like *S. daphnoides* (Sochor, Vašut, Bártová, Majeský, & Mráček, 2013) and *S. hukaona* (Kikuchi et al., 2011). These dynamics are probably more accentuated in upstream populations where erosion is more intense than downstream (Imbert & Lefévre, 2003; Kikuchi et al., 2011; Stamati, Hollingsworth, & Russell, 2007). In our case, Ameca and La Parota are upstream populations, which is congruent with this explanation.

We also found lower but significant levels of genetic structure (*F*_ST_) associated with different rivers within basins. In other Salicaceae species, lack of genetic structure is commonly attributed to the absence of geographic barriers and long‐distance dispersal of seeds and pollen through wind (Perdereau, Kelleher, Douglas, & Hodkinson, 2014). In contrast to *Populus*, *Salix* is insect pollinated, and dispersal of seeds by wind is limited to about 200 m from the source plant (Gage & Cooper, 2005). Also, as stated before, the geographical area studied has an extremely complex topography, making long‐distance wind‐mediated gene flow difficult and limiting it even among spatially close populations. In these circumstances, hydrochory could be an effective way of secondary dispersion, but restricting gene flow to populations downstream in the same system (Kikuchi et al., 2011; Pollux, Luteijn, Groenendael, & Ouborg, 2009; Seiwa et al., 2008).

### 4.3 Ecological differentiation

Both cpDNA and nuclear markers indicated a strong differentiation and low gene flow among populations situated in different basins. As previously explained, this differentiation could be related to the historical and current topographical configuration of the basins. However, it is also possible that environmental differences are also contributing to limit gene flow. To explore this idea, we performed niche differentiation tests among basins and determined whether genetic differentiation is related to environmental distances more than to spatial distances.

Our results indicated that slope and precipitation of the warmest quarter (Bio 18) were highly informative for both Balsas and Lerma, while precipitation of the wettest quarter (Bio 16) was highly informative for the Balsas model and temperature seasonality (Bio 4) was informative for the Lerma model. Considering the differentiation of niches, the combination of a high proportion of overlap (54.37%–45.62%) and interpredictability (90.62%–100%) of potential distributions, and the results of the equivalency and similarity tests, in which Lerma and Balsas ecological spaces are significantly nonidentical and also significantly not similar, a complex biogeographical scenario can be proposed in which probably vicariance primarily drove the spatial isolation of *S. humboldtiana*, followed by the effect of ecological factors as a second force of differentiation among populations. It is plausible that slope and precipitation seasonality are important variables that could determine the establishment of *S. humboldtiana* in these basins of the MCP, while the differences between precipitation in the wettest period of the year and temperature seasonality could be acting as factors of differentiation between basins. The latter agrees with the ecological and environmental differences among basins: While warmer temperatures, contrasting elevations, and tropical dry forest vegetation characterize the Balsas Basin, the Lerma Basin shows more constant elevations, more temperate conditions, and mainly oak‐pine forest vegetation (Challenger, 1998).

The RDA analysis also showed an association between climate and genetic variation, with precipitation variables as the most important ones (annual precipitation, precipitation of the wettest month, and precipitation of the warmest quarter) followed by temperature mean diurnal range and slope. It has been shown that morphological and genetic variation is associated with climatic variables in other tree species like oaks (Riordan et al., 2016; Sork et al., 2016). In our case, the fact that genetic differentiation among populations is related to differences in precipitation regimes across localities seems reasonable given the importance of these variables for the establishment of willows and also probably for their reproductive phenology. As previously mentioned, slope is also determinant for the establishment of willows along river banks (Azami et al., 2013). These results are also in agreement with the ecological niche models and comparisons.

In conclusion, the complex geological and climatic history of the MCP, including the volcanic activity of the TMVB, has had a profound impact on gene flow patterns in *S. humboldtiana* and many other organisms. However, this is the first study in the MCP to examine patterns of genetic differentiation in a riparian tree that is dispersed by wind and water. We found that even for an organism like *Salix*, characterized in general by a high dispersal capacity, gene flow in this region is restricted between basins and that genetic structure is strongly mediated by river configuration, limiting genetic exchange even between close populations. These results contrast with other studies of *Salix* species in the world, which show efficient gene flow though wind‐dispersed seeds and scarce isolating barriers between populations (Perdereau et al., 2014).

## CONFLICT OF INTEREST

None declared.

## AUTHOR CONTRIBUTIONS

Mariana S. Hernández‐Leal and Daniel Piñero designed the study and collected the data. Marco Suárez‐Atilano and Mariana S. Hernández‐Leal analyzed the data and wrote the manuscript with input from all authors. All authors discussed the results and commented on the manuscript. Antonio González‐Rodríguez and Daniel Piñero supervised the project.

## Data Availability

DNA sequences: Genbank accessions MH919415–MH919426 (psbA‐trnH) and MH924999–MH925010 (5′‐trnH). nSSRs and cpSSRs Dryad repository identification https://doi.org/10.5061/dryad.vg11d5s/1
