# Supplementary material for: Regional patterns of genetic structure and environmental differentiation in willow populations (Salix humboldtiana Willd.) from Central Mexico
Source: Ecol Evol. 2019 Aug 2;9(17):9564–79. doi: 10.1002/ece3.5475 (PMC6745842; doi:10.1002/ece3.5475)

**APPENDIX S1 Methods**

**Table S1. Information of *Salix humboldtiana* sampling sites.** Location name, state and watershed (river or lake) of sample origin; **Pop ID** Population identification number; **No. of individuals** number of individuals collected and used for each genetic marker; (**#clones**) number of individuals with repeated multilocus genotypes; and geographical coordinates. Colors correspond to three main basins studied: Blue (Lerma), Green (Balsas), and Orange (Ameca).

| **Pop ID** | **Location name** | **State** | **Watershed** | **No. of individuals** | |  | **#clones** | **Geographical coordinates** | | | |
| --- | --- | --- | --- | --- | --- | --- | --- | --- | --- | --- | --- |
|  |  |  |  | **nSSR and cpSSR** | **cpDNA** |  |  | **°N** | | | **°W** |
| 1 | Charo | Michoacán | Cuitzeo | 10 | - |  | - | | 19.737 | -101.027 | |
| 2 | Queréndaro | Michoacán | Cuitzeo | 9 | - |  | 4 | | 19.879 | -100.908 | |
| 3 | Lerma | Michoacán | Lerma | 9 | 2 |  | - | | 20.352 | -102.019 | |
| 4 | Pénjamo | Michoacán | Lerma | 6 | 2 |  | - | | 20.607 | -101.412 | |
| 5 | Jiquilpan | Michoacán | Sahuayo | 11 | - |  | 3 | | 19.995 | -102.617 | |
| 6 | Sn. Juanico | Michoacán | Itzicuaro | 6 | - |  | - | | 19.83 | -102.666 | |
| 7 | Tocumbo | Michoacán | Itzicuaro | 9 | - |  | - | | 19.763 | -102.618 | |
| 8 | La Parota | Michoacán | La Parota | 6 | - |  | 1 | | 18.577 | -99.298 | |
| 9 | Yautepec | Morelos | Yautepec | 19 | 2 |  | 2 | | 18.907 | -98.99 | |
| 10 | A. Amacuzac | Edo de Méx. | Amacuzac | 19 | 2 |  | 2 | | 18.848 | -99.731 | |
| 11 | B. Amacuzac | Morelos | Amacuzac | 18 | - |  | 2 | | 18.683 | -99.382 | |
| 12 | Ameca | Jalisco | Ameca | 11 | 4 |  | 2 | | 20.563 | 103.954 | |
|  | Total |  |  | 133 | 12 |  | 16 | |  |  | |

**Table S2.** Characteristics and primer sequences for *Salix bujartica* (Sb) and *Salix arbutifolia* (Cha) nSSRs loci assayed in *S. humboldtiana*, and primers for the 5´trnS and psbA-trnH intron of the chloroplast DNA. **Size range:** refers to observed fragment lengths in *S. humboldtiana*.**T:** aligment temperature in *S. humboldtiana.* Forward primers for polymorphic loci were fluorescent labeled for scoring.

**Polymorphic in *S. humboldtiana***

| **Locus** | **Motif** | **T (^o^C)** | **Size range** | **Primer sequences** |
| --- | --- | --- | --- | --- |
| Sb 201 | (CT)_n_(CA)_n_ | 50 | 172-288 | F: CCTCTTTTTCTATTGTGGTCT^6FAM^  R: GGCATGTATTTTTACTCCAAC |
| Sb 233 | (TA)_n_(TG)_n_ | 50 | 170-246 | F: AAATTACCGTCCAACTAAAGA^NED^  R: CATTAGCCATGAACAAGTAAA |
| Cha 475 | (GT)_n_ | 50 | 130-146 | F: AGGGAATGAGAGATGGTAGAGT^VIC^  R: GGGAAGGTAAGTTGGTGTTG |
| Cha 528 | (GT)_n_ | 50 | 158-172 | F: AGGAGGAAGTCAGATTCAC ^6FAM^  R: ATTCCATTAAAAGTCAATCA |
| Cha 580 | (CT)_n_(CA)_n_ | 53 | 234-248 | F: TTCGGTCTCGGAAACCTATG^NED^  R: CCCCACAACTGCAATATC |
| Cha 591 | (CT)_n_ | 53 | 146-164 | F: CCACGCATTACAAGTATCTC^PET^  R: GCGAATTACAGCTAAGAC |
| Cha 433 | (CT)_n_ | 53 | 356-394 | F:AATGAAAGGCTCTGATGTTGTA^VIC^  R: GGCCACACTCTAGTATGC |
| Cha 437 | (GA)n | 50 | 130-154 | F: CCATCATGGGTTCCACAATTAG^PET^  R: CGGCACTAGTTAGTATCAGAG |
| 5’ - TrnS | (A)n | 50 | 181-182 | F: GATCCCGGACGTAATCCTG  R: ATCGTACCGAGGGTTCGAAT |
| psbA-trnH |  | 52 | 260-261 | F: GTTATGCATGAACGTAATGCTC  R: CGCGCATGGTGGATTCACAAT CC |
| **Tested but monomorphic in *S. humboldtiana*** | | | | |
| Sb 80 | (TC)_n_ | 53 |  | F: TAATGGAGTTCACAGTCCTCC  R: ATACAGAGCCCATTTCATCAC |
| Sb 194 | (CA)_n_ | 53 |  | F: TGTGAGATAAGATTTGTCGGT  R: CCATAAATAAAAAACGTGAAC |
| Sb 196 | (GCC)_n_ | 50 |  | F: CTGTTTCCTGCCACTATTACC  R: TATAATCTGTCTCCTTTTGGC |
| Sb 243 | (GCC)nATCATTCCCC[GCC]_n_ | 50 |  | F: ATTCCTTTCTTCATCAGTAGC  R: GACAACGCCATTCACATGACC |
| Cha 472 | (CT)_n_ | 50 |  | F: GCCTCACCAAAGAAGCATCAAC  R: AACCAATTCCGTCGTCATTA |
| **No amplification in *S. humboldtiana*** | | | | |
| Sb 24 | (TG)_n_ |  |  | F: ACTTCAATCTCTCTGTATTCT  R: CTATTTATGGGTTGGTCGATC |
| Sb 38 | (TG)_n_ |  |  | F: CCACTTGAGGAGTGTAAGGAT  R: CTTAAATGTAAAACTGAATCT |
| Sb 93 | (GT)_n_ |  |  | F: GACGCACATACACCATTACAC  R: TGTTAGAAAATTAGGCACGGA |
| Sb 199 | (TG)_n_CG(TG)_n_ |  |  | F: CTATTTGGTCTCAATCACCTT  R: CTTTACCTCAGAAAATCCAGA |
| Cha 522 | (GA)_n_ |  |  | F: AACGTAGCAAGTATAGCGA  R: ATGTGGCAGAGATGTTTACAAG |

**PCR Amplification protocols**

The PCR protocol for the amplification of nSSRs and cpSSRs consisted of an initial denaturation step of 5 min at 94 ^o^C, followed by 35 cycles of 1 min at 94 ^o^C, 1 min at 50 - 53 ^o^C, 1 min at 72 ^o^C and ending with a final extension step of 8 min at 72 ^o^C. Reactions were prepared in 12 μL volumes containing 20 ng of genomic DNA, 1 unit of Taq DNA polymerase ([GoTaq®,](https://www.promega.es/resources/protocols/product-information-sheets/g/gotaq-dna-polymerase-m300-protocol/) Promega) 0.5 - 1.5 µm of each primer, 4 mM of MgCl_2_, 0.2 µm dNTPs and 5x PCR Buffer. PCRs were performed in an ABI 2720 thermocycler. PCR products were multiplexed and analyzed on an ABI Prism 3730xl DNA sequencer (Applied Biosystems) with a Liz 500 size standard. The resulting electropherograms were analyzed using the software Peak Scanner 1.0 (Applied Biosystems).

PCR reactions for chloroplast regions psbA-trnH and 5’trnS were carried out in a total volume reaction of 25 μL, containing 1x PCR buffer (Invitrogen, Carlsbad, CA, USA), 1.5 mm MgCl_2_, 0.1 mm of each dNTP, 0.1 mm of each primer, 0.5 units of Promega Taq DNA Polymerase and 10 ng of template DNA. Purification and sequencing of the PCR products were performed at the University of Washington High Throughput Genomic Center, Seattle, WA. The sequences were aligned and edited manually using Bioedit 7.2.5 (Hall, 1999).

**APPENDIX S2 Results**

**Table S3.** Estimated frequencies of null alleles at 8 nSSR loci in 12 populations of *S. humboldtiana* in the Mexican Central Plateau according to the FreeNA software.

| Locus/  Population | Cha580 | Cha591 | Cha528 | Cha 475 | Cha 433 | Cha 437 | Sb201 | Sb233 |
| --- | --- | --- | --- | --- | --- | --- | --- | --- |
| Charo | 0 | 0.06 | 0 | 0 | 0 | 0 | 0 | 0 |
| Queréndaro | 0 | 0 | 0 | 0 | 0 | 0 | 0 | 0.17 |
| Lerma | 0.05 | 0.07 | 0 | 0.32 | 0 | 0.13 | 0 | 0 |
| Penjamo | 0 | 0 | 0 | 0.20 | 0 | 0 | 0 | 0 |
| Jiquilpan | 0.29 | 0 | 0 | 0.10 | 0 | 0 | 0 | 0 |
| Tocumbo | 0 | 0 | 0 | 0.39 | 0 | 0 | 0 | 0.13 |
|  | 0 | 0 | 0 | 0.06 | 0 | 0 | 0 | 0.09 |
| La Parota | 0 | 0 | 0 | 0 | 0 | 0 | 0.27 | 0.33 |
| Yautepec | 0 | 0 | 0.21 | 0.34 | 0 | 0.07 | 0.25 | 0.33 |
| A. Amacuzac | 0 | 0 | 0 | 0.33 | 0 | 0.03 | 0 | 0 |
| B. Amacuzac | 0.04 | 0 | 0.11 | 0.34 | 0.12 | 0.12 | 0.17 | 0 |
| Ameca | 0.05 | 0 | 0 | 0 | 0 | 0.26 | 0.13 | 0.23 |

**Table S4.** Estimated *F*_ST_ values using the method of Weir (1996), with and without the ENA correction for null alleles according to the FreeNA software.

| Locus | Fst not using ENA | Fst using ENA |
| --- | --- | --- |
| Cha580 | 0.23 | 0.23 |
| Cha591 | 0.24 | 0.24 |
| Cha528 | 0.40 | 0.38 |
| Cha 475 | 0.32 | 0.32 |
| Cha 433 | 0.29 | 0.29 |
| Cha 437 | 0.19 | 0.18 |
| SB201 | 0.19 | 0.17 |
| SB233 | 0.29 | 0.30 |
| Total | 0.22 | 0.21 |

**Table S5**. Deviance information criterion (DIC) values for the complete model (nfb) and null alleles model (nb) to detect inbreeding in the four populations of *S. humboldtiana* that showed significant deviations from Hardy-Weinberg equilibrium, using the Bayesian algorithm implemented in the INEST v2.2 software.

| Population | Dic **nfb** | Dic **nb** |
| --- | --- | --- |
| La Parota | 35.661 | 42.293 |
| Yautepec | 491.263 | 500.438 |
| B. Amacuzac | 484.488 | 483.126 |
| Ameca | 260.95 | 263.081 |

**Table S6.** Pairwise genetic differentiation among populations (designated by number) of *S. humboldtiana*. *F*_ST_ values are above diagonal and *R*_ST_ values are below the diagonal. Numbers in bold are not significant. *P* < 0.05

| ***R*_ST_/F_ST_** | **1** | **2** | **3** | **4** | **5** | **6** | **7** | **8** | **9** | **10** | **11** | **12** |
| --- | --- | --- | --- | --- | --- | --- | --- | --- | --- | --- | --- | --- |
| **1** |  | 0.142 | 0.068 | 0.072 | 0.126 | 0.154 | 0.193 | 0.231 | 0.506 | 0.315 | 0.345 | 0.262 |
| **2** | **0.044** |  | 0.050 | 0.185 | 0.093 | 0.156 | 0.236 | 0.327 | 0.506 | 0.330 | 0.366 | 0.283 |
| **3** | **0.228** | **0.206** |  | 0.104 | 0.064 | 0.130 | 0.191 | 0.272 | 0.458 | 0.309 | 0.331 | 0.261 |
| **4** | **0.004** | **0.112** | **0.112** |  | 0.186 | 0.186 | 0.214 | 0.260 | 0.556 | 0.349 | 0.390 | 0.290 |
| **5** | 0.251 | 0.243 | **0.026** | 0.168 |  | 0.133 | 0.165 | 0.301 | 0.469 | 0.310 | 0.338 | 0.291 |
| **6** | 0.63 | 0.648 | 0.434 | 0.580 | 0.274 |  | 0.150 | 0.242 | 0.423 | 0.326 | 0.355 | 0.309 |
| **7** | 0.62 | 0.628 | 0.443 | 0.581 | 0.229 | **0.021** |  | 0.195 | 0.524 | 0.300 | 0.297 | 0.277 |
| **8** | 0.611 | 0.535 | 0.434 | 0.569 | 0.341 | 0.332 | **0.230** |  | 0.487 | 0.372 | 0.378 | 0.329 |
| **9** | 0.371 | 0.224 | 0.258 | 0.343 | 0.265 | 0.390 | 0.305 | **0.097** |  | 0.476 | 0.543 | 0.530 |
| **10** | 0.798 | 0.751 | 0.732 | 0.783 | 0.712 | 0.719 | 0.683 | 0.397 | 0.305 |  | 0.036 | 0.126 |
| **11** | 0.846 | 0.815 | 0.798 | 0.842 | 0.781 | 0.798 | 0.779 | 0.523 | 0.327 | **-0.01** |  | 0.098 |
| **12** | 0.403 | 0.476 | 0.358 | 0.372 | 0.153 | 0.339 | 0.297 | 0.506 | 0.433 | 0.785 | 0.841 |  |

**Niche model**

**Fig. S1**. **Ecological niche models (ENMs) obtained with MAXENT for *Salix humboldtiana*** reciprocally projected between basins: Lerma model (blue prediction) into Balsas area (green outline) and Balsas (green prediction) into Lerma (blue outline). For a comprehensive comparison see Fig. 4. Both predictions indicated the logistic probability of environmental suitability. Darker areas represent higher probability of species occurrence. Circles and squares represent unique records of *S. humboldtiana* for the two basins obtained from REMIB data. ENMs were obtained using record data that were at least 10 km separated from each other (Balsas=24), (Lerma=32).


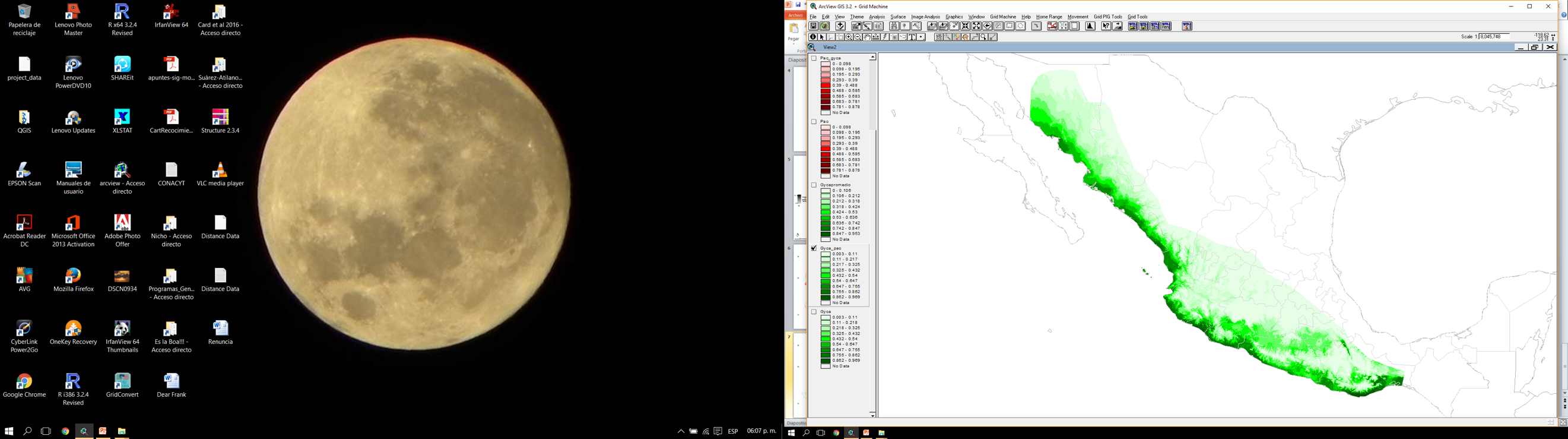


0.0-0.1

0.1-0.2

0.2-0.3

0.3-0.4

0.4-0.5

0.4-0.6

0.7-0.8

0.8-0.9

0.9-1.0

0.0-0.1

0.1-0.2

0.2-0.3

0.3-0.4

0.4-0.5

0.4-0.6

0.7-0.8

0.8-0.9

0.9-1.0


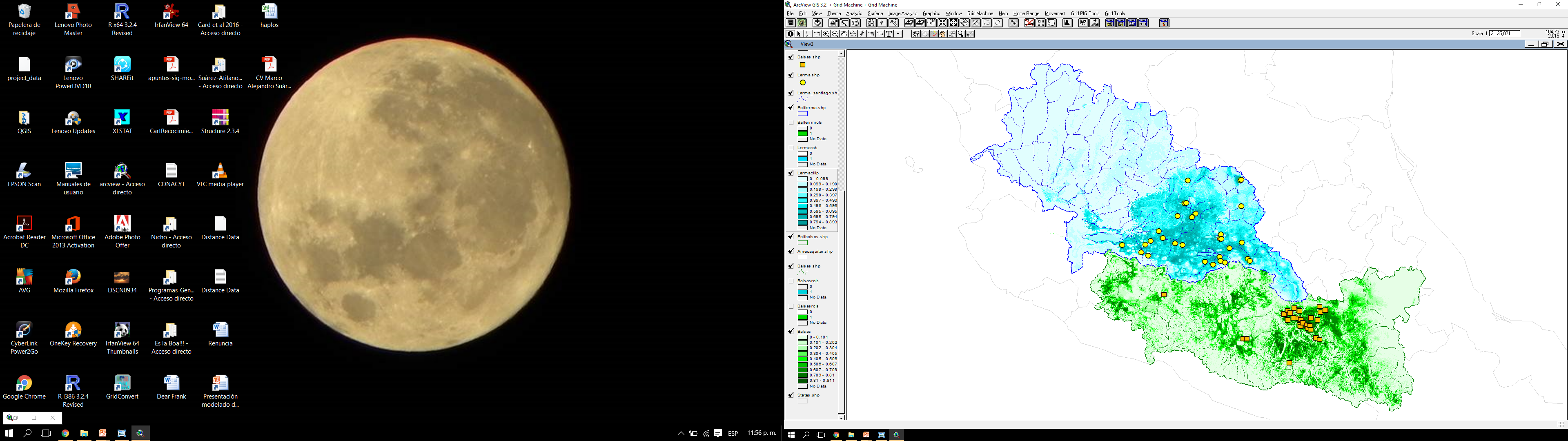


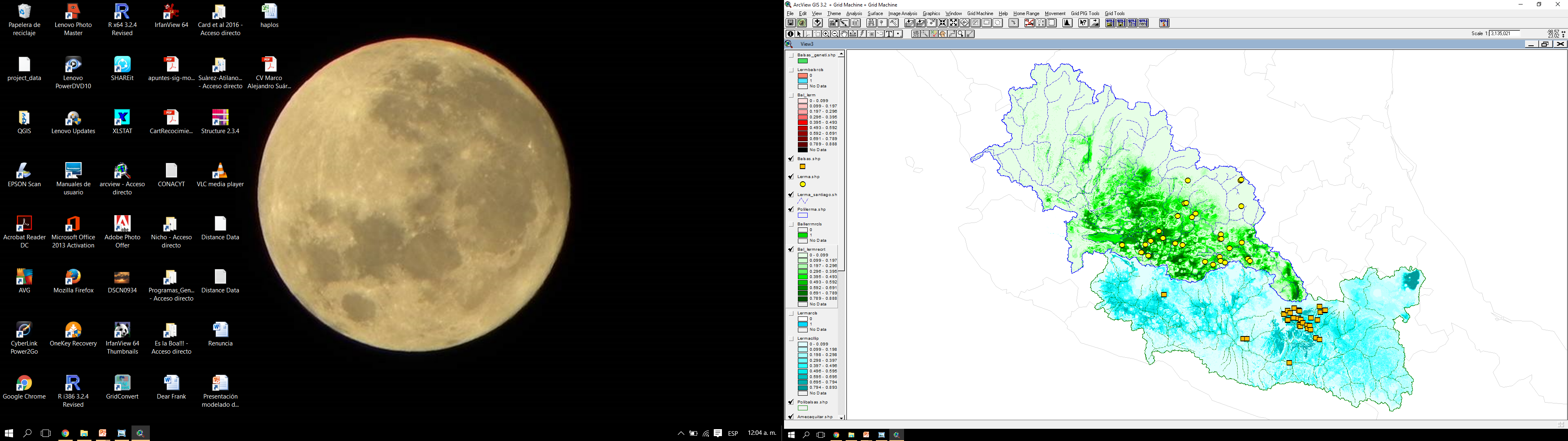

Supplement: Supplementary file 1 [file ECE3-9-9564-s001.docx]
